# Accurate Path Loss Prediction Using a Neural Network Ensemble Method

**DOI:** 10.3390/s24010304

**Published:** 2024-01-04

**Authors:** Beom Kwon, Hyukmin Son

**Affiliations:** 1 Division of Interdisciplinary Studies in Cultural Intelligence, Dongduk Women’s University, Seoul 02784, Republic of Korea; bkwon@dongduk.ac.kr; 2 Department of Electronic Engineering, Gachon University, Seongnam 13120, Republic of Korea

**Keywords:** artificial intelligence, ensemble learning, deep learning, machine learning, neural network ensemble, path loss prediction

## Abstract

Path loss is one of the most important factors affecting base-station positioning in cellular networks. Traditionally, to determine the optimal installation position of a base station, path-loss measurements are conducted through numerous field tests. Disadvantageously, these measurements are time-consuming. To address this problem, in this study, we propose a machine learning (ML)-based method for path loss prediction. Specifically, a neural network ensemble learning technique was applied to enhance the accuracy and performance of path loss prediction. To achieve this, an ensemble of neural networks was constructed by selecting the top-ranked networks based on the results of hyperparameter optimization. The performance of the proposed method was compared with that of various ML-based methods on a public dataset. The simulation results showed that the proposed method had clearly outperformed state-of-the-art methods and that it could accurately predict path loss.

## 1. Introduction

A cellular network typically comprises multiple base stations, with each mobile station measuring the received signal strength indicator from its neighboring base stations and transmitting this information to the base stations via radio signals [1,2,3]. Path loss is a phenomenon in which the strength of a radio signal between a base station and mobile station decreases as it propagates through space. Predicting path loss is crucial in base-station positioning, because mobile stations require a minimum received signal power level to successfully decode the data received from the base station [4,5,6].

Recently, several path loss models have been proposed. Generally, these models can be classified into two groups: empirical and deterministic. Empirical models are based on the measurements obtained within a given frequency range in a specific propagation environment. These models offer statistical descriptions of how path loss is related to propagation parameters, including frequency of transmission, distance between antennas, and antenna height. For example, the log-distance path loss model employs a path loss exponent empirically determined from measurements to define the rate at which the received signal strength diminishes with the distance between a base station and mobile station [7]. Additionally, a Gaussian random variable with a mean of zero is used in the model to represent the attenuation attributed to shadow fading. This model is commonly used as a fundamental reference for predicting indoor path loss. Several empirical models, including the Egli [8], Hata [9], Longley-Rice [10], and Okumura [11] models, have been developed based on measurements. The 3rd Generation Partnership Project (3GPP) is a collaborative initiative aimed at developing global standards for mobile communication technologies. The 3GPP is responsible for specifying technologies such as Long-Term Evolution (LTE) and New Radio (NR) for mobile broadband communication. The standards set by the 3GPP are continuously evolving to meet the demands of the industry and users. Organized as Releases, these standards introduce new features, enhancements, and optimizations, with each release incorporating hundreds of individual technical specification (TS) and technical report (TR) documents. Some TR documents focus on empirical models and modeling methods. For instance, in TR 38.901 [12], 3GPP introduced its three-dimensional (3-D) stochastic channel model for 5G mmWave massive multiple-input and multiple-output (MIMO) communications, spanning the range of 0.5-100 GHz. This comprehensive model includes a detailed procedure for generating link-level channel models, catering to a broad spectrum of carrier frequencies. The 3GPP employs various scenario settings, including Indoor Factory (InF), Indoor Hotspot (InH), Rural Macro (RMa), Urban Macro (UMa), and Urban Micro (UMi). Additionally, each scenario is accompanied by a comprehensive set of parameters, covering intersite distance, path loss computation, large and small-scale parameters, etc., [13,14]. Empirical models are simple and tractable and require few parameters. However, because their parameters fit the measurements obtained in a specific propagation environment, these models do not always yield high prediction accuracies when applied to diverse environments.

Regarding deterministic models, they are based on the electromagnetic theory. These models offer precise path loss values at any given position using ray tracing and finite-difference time-domain methods [15,16,17]. However, these models require detailed geometric data, such as a two-dimensional (2-D) or 3-D map of a specific region and the dielectric properties of obstacles to predict path loss. Additionally, because such data are generally large in volume, handling them can potentially result in a degraded computational efficiency and an extended computation time. Furthermore, if the propagation environment changes, a time-consuming computational procedure needs to be repeated.

Recently, machine learning (ML) has received considerable attention as a powerful tool in various fields, such as computer vision [18,19,20,21,22,23,24], natural language processing [25,26,27], and wireless communication [28,29,30,31,32]. Generally, ML approaches can be divided into three categories: supervised, unsupervised, and reinforcement learning. In supervised learning, data pairs of (x,y) are given, where *x* represents the input data of an ML model, and *y* represents a label. Supervised learning is performed to enable an ML model to learn a general rule that maps *x* to *y*. On the other hand, in unsupervised learning, only *x* is provided to the ML model; *y* is not provided. Unsupervised learning is performed to enable the model to discover hidden structures or patterns in *x*. In reinforcement learning, an agent learns to make decisions by interacting with an environment. The learning process in reinforcement learning involves the agent interacting with the environment, observing the outcomes of its actions, and adjusting its strategy to improve its performance over time. The agent learns to associate states with actions that lead to higher rewards and, through exploration, discovers optimal policies for achieving its goals. Reinforcement learning has been used for power control of base stations, scheduling, load balancing, and many more applications in wireless networks [33,34,35,36,37].

Recently, numerous supervised-learning algorithms have been introduced. These algorithms can be categorized into classification and regression algorithms based on the type of *y*. When *y* can take on values in a finite set, classification algorithms whose outputs are constrained to this finite set are employed. Conversely, if *y* can take any real value within a range, regression algorithms whose outputs are not limited to any specific real value are used. Since the value of path loss can be represented as a real value, path loss prediction can be regarded as a regression problem. Consequently, some studies on path loss prediction have proposed applying regression algorithms, such as the support vector machine (SVM), *k*-nearest neighbors (*k*-NN), random forest (RF), and artificial neural network (ANN), to predict path loss values. For example, the authors of [38,39] experimentally showed that ML-based models could predict path loss more accurately than empirical models could and that they were more computationally efficient than deterministic models. Based on these results, many researchers have focused on ML-based models as potential substitutes for conventional empirical and deterministic models. Motivated by these findings, we investigated an ML-based method to enhance the accuracy of path loss prediction. The main contributions of this study are summarized as follows:

- A neural network ensemble model capable of accurately predicting path loss is proposed. In the proposed model, multiple ANNs are trained with different hyperparameters, including the number of hidden layers, number of neurons in each hidden layer, and type of activation function, thereby enhancing the diversity among the integrated ANNs. The final prediction results of the model were then obtained by integrating the prediction results from the ANNs.
- The entire process of predicting path loss using the proposed method is presented. The dataset splitting, feature scaling, and hyperparameter optimization processes have been detailed. Based on the results of the hyperparameter optimization process, the top-ranking ANNs can be determined. These results and the pseudocode for the proposed method can simplify re-implementation.
- The proposed neural network ensemble model was quantitatively evaluated on a public dataset. Additionally, for benchmarking, nine ML-based path loss prediction methods were tested: SVM, *k*-NN, RF, decision tree, multiple linear regression, Least Absolute Shrinkage and Selection Operator (LASSO), ridge regression, Elastic Net, and ANNs.

The remainder of this paper is organized as follows. Section 2 reviews related studies, and Section 3 describes the proposed method for path loss prediction. Then, Section 4 details the experimental setup, including the evaluation metrics and implementation of the benchmark methods, and Section 5 presents and discusses the results. Finally, Section 6 provides the concluding remarks.

## 2. Related Work

### 2.1. Non-ANN-Based Path Loss Prediction

As previously mentioned, many different regression algorithms can predict path loss. Based on prior research, these algorithms can be classified into two groups: non-ANN-based and ANN-based. In a study on path loss prediction using non-ANN-based approach, a path loss equation consisting of several constants and simple functions was proposed [40]. Additionally, using a genetic algorithm, the authors identified constants and functions that fit the measurements well.

Instead of using a genetic algorithm, some researchers used an SVM for path loss prediction [41,42,43,44,45,46,47]. The authors of [41] proposed using an SVM with a radial basis function (RBF) kernel as a tool for predicting path loss. Generally, several types of kernels can be used in SVMs; the performance and effectiveness of the SVM depend on the kernel type. In [42], the authors compared the performances of three SVMs with different kernels: polynomial, Gaussian, and Laplacian. Their results revealed that SVM with a Laplacian kernel had outperformed the SVM with the other two kernels. Moreover, the authors compared the three SVMs with two empirical models (the Hata and Ericsson 9999 models); all three SVMs outperformed the empirical models. Motivated by the results of [41,42], the authors of [43,44,45,46,47] also used an SVM for path loss prediction.

The authors of [46,47,48] used *k*-NN, a well-known regression algorithm, for path loss prediction. In recent studies on non-ANN-based path loss prediction, ensemble methods that use multiple ML models to achieve better performances have garnered significant attention owing to their promising results. Based on empirical evidence, it is generally observed that ensemble methods achieve better performances when their constituent models are significantly diverse [49,50]. Consequently, several ensemble methods aim to enhance the diversity among their constituent models [51,52]. To this end, the authors of [53] proposed an ensemble method that averaged the results from five different regression algorithms: *k*-NN, SVM, RF, AdaBoost, and gradient boosting. Regarding RF, it is a widely used ensemble method that constructs numerous decision trees during the training phase. Additionally, during the testing phase, it derives the average prediction of individual trees as an output [54,55]. This method has been employed for path loss prediction [45,46,47,48].

### 2.2. ANN-Based Path Loss Prediction

Instead of using non-ANN-based approaches, several researchers have explored ANN-based methods for path loss prediction. In these approaches, hyperparameter tuning is crucial for ANNs because it directly affects their performance and generalization ability. Hyperparameters are configuration settings that are external to a model and cannot be learned from data. Unlike the weights and biases of an ANN, which are learned during the training phase, hyperparameters should be set prior to training. The hyperparameters of an ANN include the learning rate, batch size, number of hidden layers, the number of neurons in each layer, activation functions, dropout rates, and regularization strength.

To determine the optimal configuration that maximizes ANN performance, many researchers have conducted hyperparameter tuning in their studies. For example, the authors of [38,56,57] explored the relationship between ANN performance and the number of layers. Their results revealed that adding depth to an ANN by increasing the number of layers would enable accurate path loss prediction. The authors of [58] experimented with the performance of an ANN by varying the number of neurons in a hidden layer while keeping the number of hidden layers constant at one. According to their results, increasing the number of neurons improved the path loss prediction performance of the ANN. In [59], the authors proposed a differential evolution algorithm to determine the optimal number of neurons in each layer of an ANN that would achieve the best performance in path loss prediction.

Generally, activation functions are used to introduce nonlinearity into ANNs. The choice of an activation function significantly impacts the performance and generalization ability of an ANN. Several types of activation functions have been developed and used in ANNs. The RBF is a widely used activation function in path loss prediction. For example, the authors of [58,60,61] proposed an RBF neural network (RBF-NN), where the RBF was used as an activation function. In [62], the authors proposed a wavelet neural network for field-strength prediction using a wavelet function as an activation function. According to their results, the prediction performance of the wavelet neural network exceeded that of the RBF-NN. Other types of activation functions such as the hyperbolic tangent (tanh) [63,64,65,66,67,68,69,70] and sigmoid functions [71,72,73,74,75] have also been used in ANNs for path loss prediction.

Recently, several ANN variations, including the convolutional neural network (CNN), have been widely used for path loss prediction. A CNN typically consists of input, hidden, and output layers, with the hidden layers comprising one or more convolutional layers. In a convolutional layer, several convolution kernels (filters) can be used, and the dot product of each convolution kernel with the input matrix of the layer is obtained to generate feature maps. A rectified linear unit (ReLU) is commonly used as an activation function in convolutional layers; the activation maps for the feature maps are obtained by applying the ReLU, and these activation maps become the inputs to the next layer. Generally, the convolutional layer is followed by a pooling layer, and the pooling layer reduces the dimensions of data by combining the outputs of neuron clusters at one layer into a single neuron in the next layer. Through convolutional and pooling layers, CNNs can detect and extract meaningful features from images. Consequently, CNNs are commonly used to solve computer vision tasks, such as image classification and image recognition.

Owing to promising results from using CNNs in computer vision tasks, CNN-based methods have emerged in studies on path loss prediction. For example, the authors of [56] proposed a CNN-based method to predict the path loss exponent from a 3-D building map; two 2-D images obtained from a 3-D building map were utilized. One image was created by mapping the height of each building to an integer value within the range of 0–255, and the other was generated by mapping the difference between the height of the transmitter from sea level and the height of the ground from sea level to an integer value within the range of 0 to 255. The two images were stacked in the form of a 3-D tensor, which was used as the input for the CNN. The CNN was trained using synthetic data generated using a ray-tracing tool to predict the path loss exponent.

Recently, some popular CNN architectures proposed for computer vision tasks have been applied to path loss prediction. For example, the authors of [76] utilized AlexNet, which was proposed in [77], as the base model for path loss prediction. The model input consisted of a 3-D tensor constructed by stacking three 2-D matrices. These matrices contained information about the height of structures and buildings, the distance from the transmitter, and the distance from the receiver. Another study by the same authors employed AlexNet as the base model [78]. In this study, the 3-D tensor was augmented with a 2-D matrix containing information about the angle formed by the line between the transmitter and receiver. The Visual Geometry Group neural network (VGGNet) [79] is another well-known CNN architecture. It can be categorized into several architectures according to the number of convolutional layers. Among them, the VGG-16 and VGG-19 architectures are typically used because their performance is better than that of other VGGNet architectures. In [80], the authors utilized the VGG-16 architecture to predict the path loss distribution from 2-D satellite images. Motivated by the idea presented in [80], the authors of [81] employed the VGG-16 architecture as the backbone to predict the path loss exponent and shadowing factor from 2-D satellite images. The residual neural network (ResNet) [82] is also a widely used CNN architecture. The ResNet architecture was used in a similar study to predict the path loss exponent and shadowing factor from 2-D satellite images [83] and in another study [84] to predict the path loss from 2-D satellite images.

## 3. Proposed Method

### 3.1. Overall Process

**Figure 1:**
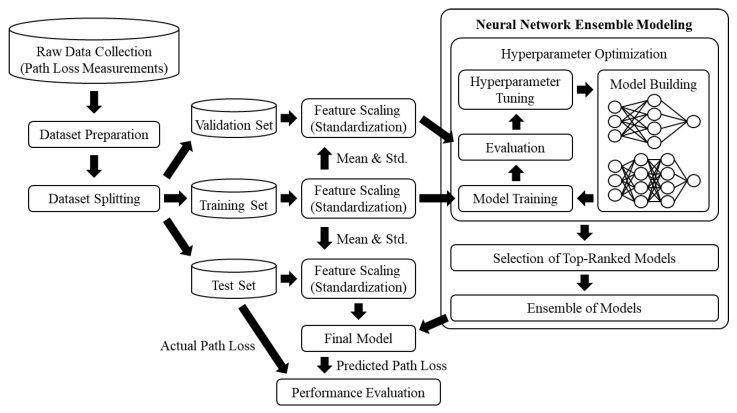
Overall working of the proposed method for path loss prediction.

This section details the working of the proposed path loss prediction method; Figure 1 shows a schematic overview of its process, which is divided into three phases: (1) dataset splitting and feature scaling, (2) model building and hyperparameter optimization, and (3) applying the ensemble model and performing path loss prediction. In the first phase, dataset splitting is conducted on the prepared dataset, producing training, validation, and test sets. Subsequently, feature scaling is applied to enhance the performance of the ANNs. In the second phase, the ANNs are built with different hyperparameter configurations; the hyperparameters include the number of hidden layers, number of neurons in each hidden layer, and type of activation function. During the hyperparameter optimization process, each ANN is trained and evaluated, and the results are recorded. In the final phase, the top-ranked ANNs are selected based on the evaluation results, and the final model is constructed using an ensemble of the selected ANNs. A path loss prediction is conducted on the test set using the final model.

### 3.2. Dataset Preparation

The dataset proposed by the authors in [85] was used in this study. To collect path loss data, these authors conducted a drive test measurement campaign at Covenant University, Ota, Ogun State, Nigeria. During the drive tests, measurements were performed along three different routes. During each measurement, the mobile station was moved away from each of the three base stations. These authors recorded terrain profile information, including longitude ($f_{1}$), latitude ($f_{2}$), elevation ($f_{3}$), altitude ($f_{4}$), clutter height ($f_{5}$), and distance between the transmitter and receiver ($f_{6}$), along with path loss data. Across the three routes, 937, 1229, and 1450 samples were collected; the dataset contained 3616 samples, comprising six features and path loss values as labels. In this study, we aimed to obtain a generalized neural network ensemble model rather than a site-specific model. To reach this goal, all 3616 samples were used without further divisions.

### 3.3. Dataset Splitting and Feature Scaling

In this study, the dataset was randomly shuffled and then split into training, validation, and testing sets. A training set was used to train the model. If the model was evaluated on the same data on which it had been trained, it might have performed well on that specific dataset but would have failed to generalize to new data (overfitting); the validation set helped detect and prevent this issue. Moreover, the validation set allowed the tuning of the hyperparameters without introducing bias from the test set. The test set provided an unbiased evaluation of the final performance of the model, indicating how well it would perform on new real-world data. Generally, separating data into training, validation, and test sets can ensure that ML models are robust, generalize well to new data, and perform reliably in real-world scenarios. More specifically, in our study, 60% of the 3616 samples were allocated to the training set, whereas 20% was assigned each to the validation and test sets.

Table 1 presents the descriptive statistics of the training dataset. As indicated in the table, the scales of the six features differed. Generally, if the features are on different scales, ML algorithms may assign greater importance to features with larger magnitudes. Additionally, these algorithms can be sensitive to the scale of the input features, thereby affecting their performance. To mitigate these issues, the features were standardized by removing the mean and scaling to the unit variance. Let $x_{j}=\left[f_{1,j},f_{2,j},f_{3,j},f_{4,j},f_{5,j},f_{6,j}\right]$ be the *j*th sample in the dataset, where $f_{1,j}$, $f_{2,j}$, $f_{3,j}$, $f_{4,j}$, $f_{5,j}$, and $f_{6,j}$ are the corresponding feature values of the *j*th sample. Then, the standard score of each feature value in $x_{j}$ is calculated as:

(1) $${\tilde{f}}_{i,j}=\frac{\left(f_{i,j}-{\bar{f}}_{i}\right)}{\sigma_{i}},\;\;\;\forall i\in \left\{1,2,3,4,5,6\right\},$$

where ${\bar{f}}_{i}$ is the mean of $f_{i}$ for the training samples, and $\sigma_{i}$ is the standard deviation of $f_{i}$ for the training samples.

### 3.4. Hyperparameter Optimization

Our proposed model consists of multiple ANNs, each of which can have multiple fully connected layers as hidden layers; every input neuron is connected to every output neuron, which is a configuration commonly used in ANNs. To construct the optimal ensemble structure, hyperparameter optimization processes were executed. The considered hyperparameters included the number of hidden layers, number of neurons in each hidden layer, and type of activation function. Throughout these processes, a training dataset was used to train each ANN. Early stopping was applied to prevent the training of the ANN for an excessive number of epochs, which could lead to overfitting; a validation dataset was used to detect and prevent overfitting. For each hyperparameter configuration, the mean squared error (MSE) of the ANN was computed for the validation dataset.

**Figure 2:**
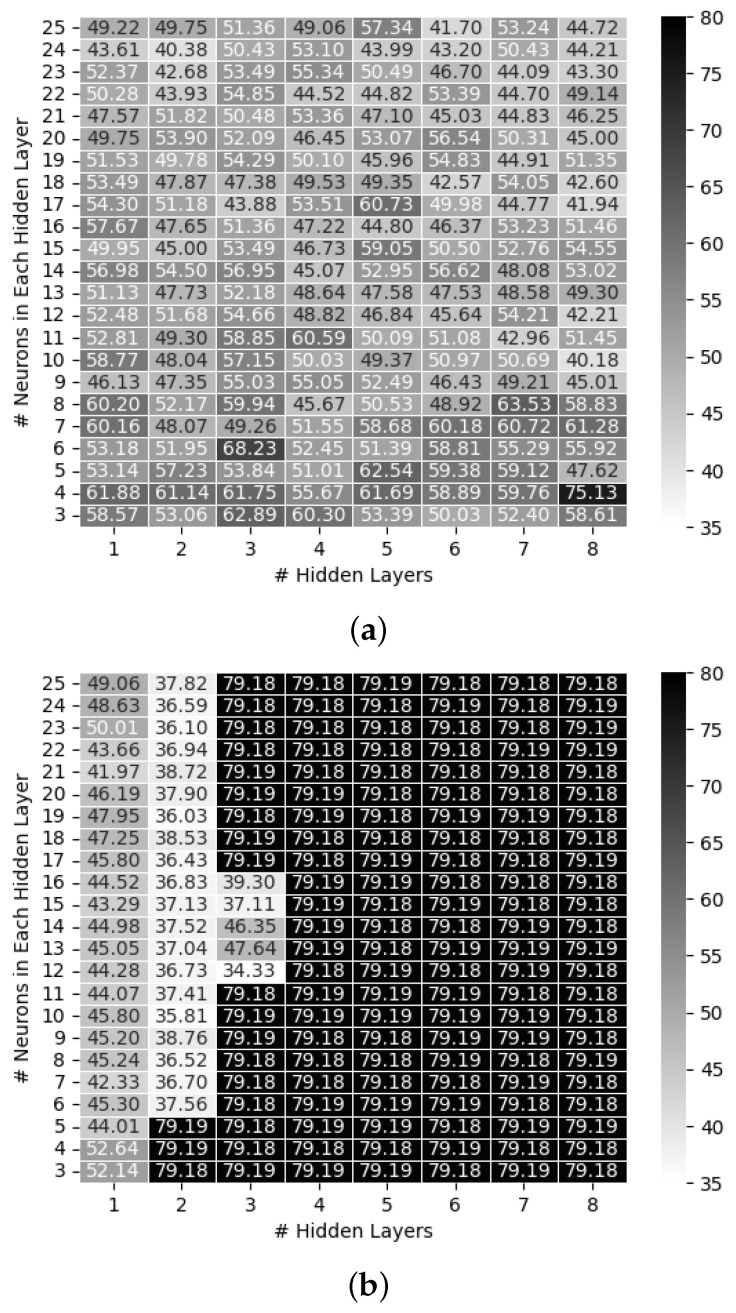

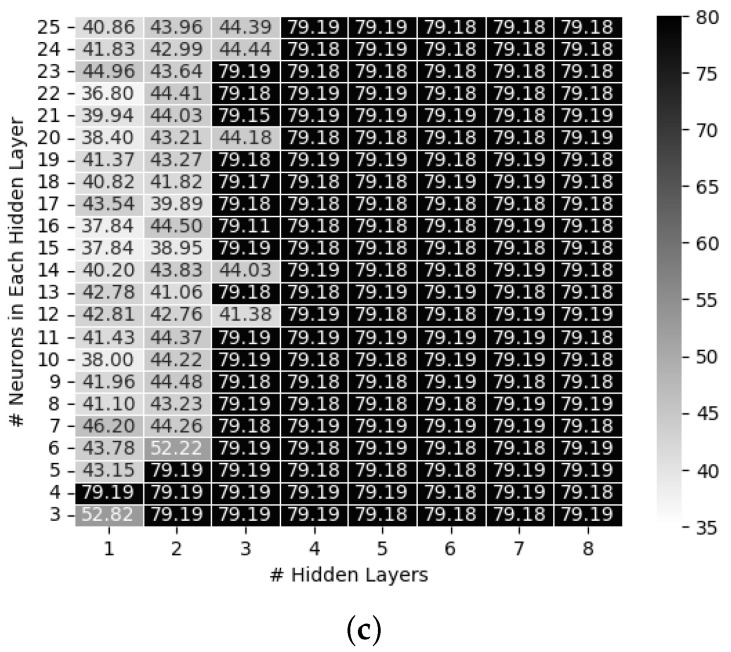
Heat map of MSE values based on *M* and *N*: (**a**) ReLU, (**b**) sigmoid, and (**c**) tanh.

For clarity, let *M* be the number of hidden layers in the ANN and *N* be the number of neurons in each hidden layer. Figure 2 shows a heat map of the MSE values based on *M* and *N*. In the experiments shown in Figure 2a, the ReLU was used as the activation function in each hidden layer, and the ANN with M=8 and N=10 achieved the minimum MSE; in those shown in Figure 2b, a sigmoid function was used as the activation function and an ANN with M=3 and N=12 achieved the minimum MSE; and in those shown in Figure 2c, a hyperbolic tangent function was used, and the ANN with M=1 and N=22 achieved the optimal MSE. The results presented in Figure 2b,c showed that the MSE of the ANNs had never decreased below 79 for *M* values ≥4.

### 3.5. Ensemble of Artificial Neural Networks

As illustrated in Figure 1, the proposed method involves a neural network ensemble model composed of multiple ANNs. To enhance the diversity among the integrated ANNs, the top *T* ANNs were selected based on the results of the hyperparameter optimization. For simplicity, hereafter, the selected top *T* ANNs shall be referred to as $ANN_{1}$, $ANN_{2}$, $ANN_{3}$, ⋯, $ANN_{T-1}$, and $ANN_{T}$. Table 2 lists the hyperparameter configurations and MSE of the 20 highest-ranking ANNs.

The pseudocode for the proposed neural network ensemble method is presented in Algorithm 1. As shown in the pseudocode, the given dataset was split into training, validation, and test sets. Feature scaling was applied to each set using Equation (1). Subsequently, various ANNs were constructed with different hyperparameter configurations. Each ANN was trained using a training dataset and evaluated on the validation dataset, and the MSE results were recorded. The top-ranked ANNs were selected based on their MSE results, and the final model was constructed using an ensemble of the selected ANNs.
**Algorithm 1** Pseudocode for the proposed neural network ensemble method**Input:** 
Dataset *D*
**Output:** 
Final ensemble model *E*
1:Split *D* into training, validation, and test sets ($D_{training}$, $D_{validation}$, and $D_{test}$, respectively)2:Determine ${\bar{f}}_{i}$ and $\sigma_{i}$3:Apply feature scaling to $D_{training}$, $D_{validation}$, and $D_{test}$ using Equation (1)4:Set *T*, *M*, and *N*5:Set the maximum epochs (max_epochs)6:Set the number of training samples in the batch (batch_size)7:Create an early stopping callback (es_cb)8:Create an empty list H9:**for** m=0 to *M* **do**10:   **for** n=1 to *N* **do**11:       **for** activation in {“ReLU”, “sigmoid”, “tanh”} **do**12:          model = Build_Neural_Network(*m*, *n*, activation)13:          model = Training(model, $D_{training}$, $D_{validation}$, max_epochs, batch_size, es_cb)14:          val_loss = Evaluation(model, $D_{validation}$)15:          Append [val_loss,model] to H16:       **end for**17:   **end for**18:**end for**19:Sort each model in ascending order based on the val_loss recorded on H20:Select the top-ranked *T* models21:*E* = Build_Ensemble(selected *T* models)22:**return** *E*


The key concept behind the proposed neural network ensemble model was training multiple ANNs with different subsets of hyperparameters and aggregating their predictions. Through this process, the proposed model became more robust and less prone to overfitting. The ensemble nature of the model helped improve the generalization and predictive performance. During the prediction phase, each ANN in the ensemble independently predicted input data. The predictions from all ANNs were then aggregated to produce the final prediction. In this study, the final output of the neural network ensemble model was the average of the predictions made by each ANN. For clarity, let ${\hat{y}}_{r}$ be the path loss value predicted by $ANN_{r}$. Then, the predicted path loss values from *T* ANNs can be represented as vector $\hat{Y}$ as follows:

(2) $$\hat{Y}=\left[{\hat{y}}_{1},{\hat{y}}_{2},\cdots ,{\hat{y}}_{T-1},{\hat{y}}_{T}\right].$$

To derive the final prediction result from $\hat{Y}$ in Equation (2), the predictions of *T* ANNs were averaged.

## 4. Experimental Setup

### 4.1. Evaluation Metrics

Generally, using multiple metrics in the performance evaluation of algorithms provides a more comprehensive and nuanced understanding of their performance; relying on a single metric may result in an incomplete or biased assessment. Therefore, for our performance evaluation, we utilized various metrics, including the MSE, root mean square error (RMSE), mean absolute error (MAE), mean absolute percentage error (MAPE), mean squared logarithmic error (MSLE), root mean squared logarithmic error (RMSLE), and coefficient of determination. For clarity, let $y_{j}$ be the actual path loss value for the *j*th sample $x_{j}$ in the dataset, ${\bar{y}}_{j}$ be the predicted path loss value for $x_{j}$, and *S* be the total number of ${\bar{y}}_{j}$ generated from *S* samples in the dataset. Subsequently, the MSE, which measures the average of the squares of the errors, can be computed using the following formula:

(3) $$MSE=\frac{1}{S}\sum_{j=1}^{S}{\left(y_{j}-{\bar{y}}_{j}\right)}^{2}.$$

Although the RMSE and MSE are similar in terms of model scoring, they are not always immediately interchangeable, with MSE tending to be more sensitive to outliers, treating all errors equally regardless of their magnitude. If outliers are present in the dataset, MSE may be influenced more by these extreme values. In contrast, the RMSE tends to be less sensitive to outliers because it involves the square root of the squared errors; the RMSE is defined as the square root of the MSE, as follows:

(4) $$RMSE=\sqrt{MSE}=\sqrt{\frac{1}{S}\sum_{j=1}^{S}{\left(y_{j}-{\bar{y}}_{j}\right)}^{2}}.$$

Compared with the MSE, the MAE is less sensitive to outliers because each error term contributes equally to the overall error because it is based on absolute differences; the MAE is defined as the average of the absolute errors:

(5) $$MAE=\frac{1}{S}\sum_{j=1}^{S}\left|y_{j}-{\bar{y}}_{j}\right|.$$

Regarding the MAPE, it is defined as follows:

(6) $$MAPE=\frac{1}{S}\sum_{j=1}^{S}|\frac{y_{j}-{\bar{y}}_{j}}{y_{j}}|.$$

Then, the MSLE, which measures the mean of the squared logarithmic differences between $y_{j}$ and ${\bar{y}}_{j}$, can be computed as follows:

(7) $$MSLE=\frac{1}{S}\sum_{j=1}^{S}{\left\{\log\left(y_{j}+1\right)-\log\left({\bar{y}}_{j}+1\right)\right\}}^{2}.$$

Concerning the RMSLE, it is defined as the square root of the MSLE, as follows:

(8) $$RMSLE=\sqrt{MSLE}=\sqrt{\frac{1}{S}\sum_{j=1}^{S}{\left\{\log\left(y_{j}+1\right)-\log\left({\bar{y}}_{j}+1\right)\right\}}^{2}}.$$

The coefficient of determination, which is denoted by $R^{2}$, is defined as follows:

(9) $$R^{2}=1-\frac{\sum_{j=1}^{S}{\left(y_{j}-{\bar{y}}_{j}\right)}^{2}}{\sum_{j=1}^{S}{\left(y_{j}-\bar{y}\right)}^{2}},$$

where $\bar{y}$ is the mean of the actual path loss values in the dataset (i.e., $\bar{y}=1/S\times \sum_{j=1}^{S}y_{j}$), and $R^{2}$ is typically used as a measure of the goodness-of-fit of a model, with an $R^{2}$ value of 1 indicating that the predictions of the model fit the actual data perfectly.

### 4.2. Implementation of Benchmark Methods

In our experiments, to compare the performance of the proposed method, we implemented nine path loss prediction methods: (1) SVM-based, (2) *k*-NN-based, (3) RF-based, (4) decision tree (DT)-based, (5) multiple linear regression (MLR)-based, (6) LASSO-based, (7) ridge regression-based, (8) Elastic Net-based, and (9) ANN-based methods. To achieve this, the scikit-learn ML library for Python was utilized. The optimal hyperparameter configuration for each model was determined using the HalvingGridSearchCV class. The nine methods are detailed below.

#### 4.2.1. SVM-Based Path Loss Prediction Method

An SVM was employed for path loss prediction in [41,42,43,44,45,46,47]. In our study, an SVM was implemented using an SVR class in the scikit-learn library. The SVR class is an implementation of epsilon-support vector regression and includes various hyperparameters, such as the kernel type, kernel coefficient, and regularization parameter. Table 3 presents the optimal hyperparameter combinations for SVM, as determined through the hyperparameter optimization process.

#### 4.2.2. *k*-NN-Based Path Loss Prediction Method

The *k*-NN was employed for path loss prediction in [46,47,48]. It was implemented using the KNeighborsRegressor class in the scikit-learn library. This class includes various hyperparameters such as the number of neighbors, type of weight function used in the prediction, and type of metric used for distance computation. Table 4 presents the optimal hyperparameter combination for *k*-NN, as determined using the hyperparameter optimization process.

#### 4.2.3. RF-Based Path Loss Prediction Method

The RF technique was employed for path loss prediction in [45,46,47,48]. In our study, it was implemented using the RandomForestRegressor class in the scikit-learn library. This class includes various hyperparameters, such as the number of decision trees in the model, the type of function used to measure the quality of a split, and the maximum depth of the tree. Table 5 presents the optimal hyperparameter combination for RF, as determined through the hyperparameter optimization process.

#### 4.2.4. DT-Based Path Loss Prediction Method

A DT predicts a continuous value by recursively partitioning the data based on the input features and creating a tree structure in which each leaf node contains the predicted value for instances that follow the path to that leaf. It can also be employed for path loss prediction. In our study, DT was implemented using the DecisionTreeRegressor class in the scikit-learn library. This class includes various hyperparameters, such as the type of function used to measure the quality of a split, the strategy used to choose the split at each node, and the maximum depth of the tree. Table 6 presents the optimal hyperparameter combination for DT, as determined through the hyperparameter optimization process.

#### 4.2.5. MLR-Based Path Loss Prediction Method

Multiple Linear Regression (MLR) is an extension of simple linear regression, which models the relationship between a dependent variable and multiple independent variables. In a simple linear regression, there is only one independent variable, whereas in multiple linear regression, there are two or more independent variables. The coefficients of the independent variables and the y-intercept are estimated from the training samples using methods such as the least-squares method, which minimizes the MSE. Regarding MLR, it is widely used in various fields to predict outcomes, understand the relationships between variables, and determine the strength and significance of these relationships. In our study, MLR was implemented using the LinearRegression class in the scikit-learn library and employed as a benchmark method. Table 7 presents the optimal hyperparameter combination for MLR, as determined by the hyperparameter optimization process.

#### 4.2.6. LASSO-Based Path Loss Prediction Method

The LASSO regularization technique is used in linear regression to prevent overfitting and encourage simpler models. Linear regression is performed to determine the coefficients of the independent variables that best fit the observed data. Regarding LASSO, it introduces a penalty term for the traditional linear regression objective function. The penalty term, denoted by L1, is proportional to the absolute values of the coefficients. For brevity, the linear regression model trained with L1 was named LASSO. LASSO was implemented using the LASSO class in the scikit-learn library and employed as a benchmark method. Table 8 presents the optimal hyperparameter combination for LASSO, as determined through the hyperparameter optimization process.

#### 4.2.7. Ridge-Based Path Loss Prediction Method

Ridge regression, also known as Tikhonov regularization, is a technique used in linear regression to address multicollinearity and prevent overfitting. Like LASSO, ridge regression introduces a penalty term to the traditional linear regression objective function. The penalty term, denoted by L2, is proportional to the square of the coefficient. Unlike LASSO, ridge regression does not force the coefficients to be zero. Instead, it reduces the coefficients toward zero, thereby reducing their magnitudes. Advantageously, ridge regression can handle multicollinearity, which occurs when the independent variables in a regression model are highly correlated. Multicollinearity can also lead to unstable and unreliable coefficient estimates. The ridge regression adds a penalty term to the objective function, which mitigates the impact of multicollinearity by discouraging overly large coefficients. For brevity, the linear regression model trained with L2 was named Ridge. It was implemented using the Ridge class in the scikit-learn library and employed as a benchmark method. Table 9 presents the optimal hyperparameter combination for MLR, as determined through the hyperparameter optimization process.

#### 4.2.8. Elastic Net-Based Path Loss Prediction Method

Elastic Net is a regularization technique used in linear regression that combines both the L1 (LASSO) and L2 (Ridge) regularization penalties. It is designed to find a balance between LASSO and Ridge regression and thereby overcome some of their limitations. Elastic Net introduces a new hyperparameter that controls the combination of L1 and L2 penalties. The regularization term in the Elastic Net is a linear combination of both penalties. In this study, an Elastic Net was implemented using the ElasticNet class in the scikit-learn library and used as the benchmark method. Table 10 presents the optimal hyperparameter combinations for the Elastic Net determined through the hyperparameter optimization process.

#### 4.2.9. ANN-Based Path Loss Prediction Method

As previously noted, ANNs have been employed for path loss prediction [38,56,57,58,59,60,61,62,63,64,65,66,67,68,69,70,71,72,73,74,75]. Based on the details of hyperparameter configurations for ANNs in existing research, ANNs were implemented in our study using the TensorFlow library and employed as benchmarks. Table 11 lists the hyperparameter combinations for the ANNs.

## 5. Results and Discussion

Table 12 lists the performance metrics for the ensemble models with different numbers of ANNs; it shows that the best performance of the proposed ensemble model was when T=20. Therefore, in this study, the optimal value of *T* was set as 20. In other words, the proposed ensemble model consisted of 20 $ANN_{r}$ models (r∈{1,2,⋯,19,20}). Clearly, the performance of the ensemble model increased as the number of ANNs increased. However, when the number of ANNs exceeded 20, the performance of the ensemble model did not improve further. Based on these results, an ensemble model consisting of 20 ANNs was selected as the final model.

Table 13 lists the MSE, RMSE, MAE, MAPE, MSLE, RMSLE, and $R^{2}$ of each path loss prediction method. Clearly, the proposed ensemble model performed the best across all evaluation metrics. This was because the ensemble model comprised the top-ranked ANNs selected based on the MSE results, thereby enhancing the diversity among the integrated ANNs and enabling the model to achieve a robust and accurate path loss prediction performance. Among the considered benchmark methods, the *k*-NN-based method achieved the best performance, whereas the ANN-based method with the hyperparameters described in [38] achieved the worst performance. The MAE of the proposed method was approximately 1.2753, whereas that of the *k*-NN-based method was 2.4983. The MAE of the proposed method was approximately 1.223 less than that of the *k*-NN-based method. The results in Table 13 revealed that the proposed method could predict path loss accurately.

**Figure 3:**
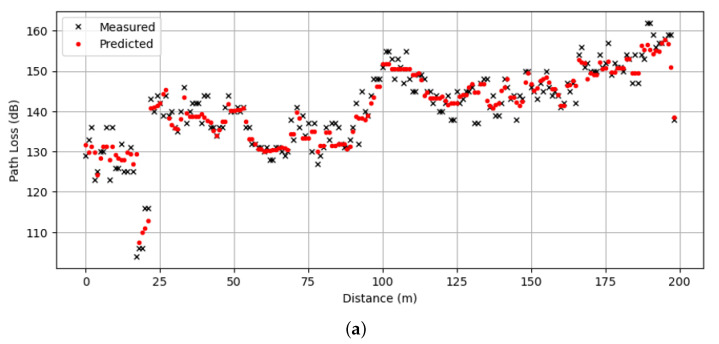

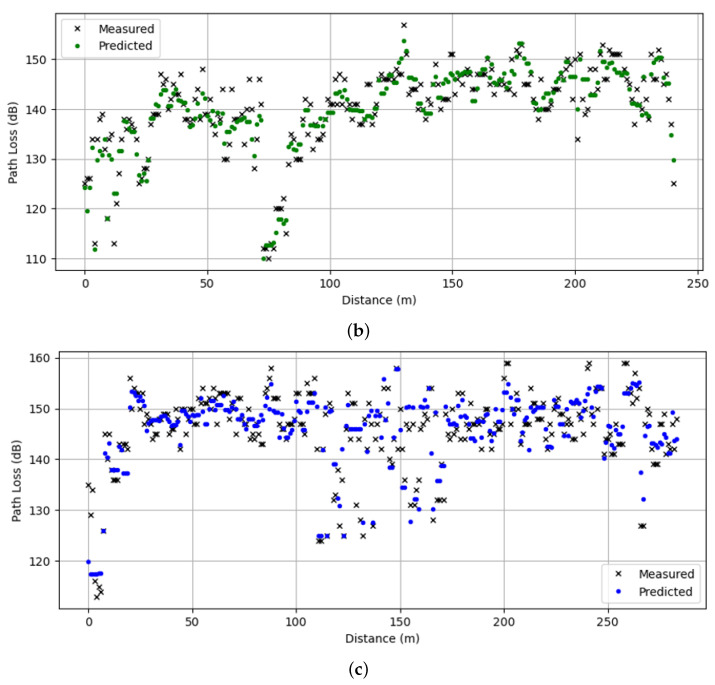
Measured and predicted path loss against distance along three survey routes: (**a**) Survey Route X, (**b**) Survey Route Y, and (**c**) Survey Route Z.

Figure 3 shows the measured and predicted path loss for three survey routes. In the figure, the values of the measured path loss data were plotted against the corresponding distance. To achieve this, we sorted the data in the test set by the distance between the transmitter and receiver after splitting the test set according to the survey route. In all survey routes, the receiver encountered non-line-of-sight (NLoS) conditions, attributed to obstructions such as buildings and trees. From the figure, it is seen that the prediction performance of the proposed method aligns closely with the measured data. This result is consistent with the performance shown in Table 13.

## 6. Conclusions

In this study, we propose a novel ML-based method for path loss prediction. Our approach leveraged the power of neural network ensemble learning and provided a robust and accurate prediction model. By constructing an ensemble of neural networks and selecting the top-ranked networks based on a hyperparameter optimization process, the method achieved a state-of-the-art performance in path loss prediction, as evidenced by the results of rigorous validation on a publicly available dataset. Furthermore, we comprehensively compared its performance with that of various ML-based methods. The simulation results demonstrated the superior performance of the proposed method. Future research directions may explore fine tuning the model, considering additional parameters, and expanding the dataset to ensure the generalizability of the proposed method across diverse scenarios.

## Figures and Tables

**Table 1:** Descriptive statistics of the training dataset.

	Longitude	Latitude	Elevation (m)	Altitude (m)	Clutter Height (m)	Distance (m)	Path Loss (dB)
Count	2169	2169	2169	2169	2169	2169	2169
Mean	3.1638	6.6745	54.39	54.80	5.81	443.83	143.18
Std	0.0038	0.0025	5.89	3.91	2.77	270.23	9.21
Min	3.1559	6.6676	45.00	49.00	4.00	2.00	104.00
25%	3.1606	6.6730	49.00	52.00	4.00	250.00	139.00
50%	3.1634	6.6745	54.00	54.00	6.00	384.00	145.00
75%	3.1670	6.6757	59.00	57.00	6.00	668.00	149.00
Max	3.1706	6.6789	64.00	64.00	16.00	1132.00	162.00

Count: Number of samples; Std: standard deviation; 25, 50, and 75% indicate the 25th, 50th, and 75th percentiles, respectively.

**Table 2:** Hyperparameter configuration and MSE for the 20 highest-ranked ANNs.

Rank	# Hidden Layers (*M*)	# Neurons in Each Hidden Layer (*N*)	Activation Function	Mean Squared Error (MSE)
1	3	12	sigmoid	34.33
2	2	10	sigmoid	35.81
3	2	19	sigmoid	36.03
4	2	23	sigmoid	36.10
5	2	17	sigmoid	36.43
6	2	8	sigmoid	36.52
7	2	24	sigmoid	36.59
8	2	7	sigmoid	36.70
9	2	12	sigmoid	36.73
10	1	22	tanh	36.80
11	2	16	sigmoid	36.83
12	2	22	sigmoid	36.94
13	2	13	sigmoid	37.04
14	3	15	sigmoid	37.11
15	2	15	sigmoid	37.13
16	2	11	sigmoid	37.41
17	2	14	sigmoid	37.52
18	2	6	sigmoid	37.56
19	2	25	sigmoid	37.82
20	1	15	tanh	37.83

**Table 3:** Hyperparameter optimization results for SVM.

Hyperparameter	Search Range	Determined Value
kernel	{“linear”, “poly”, “rbf”, “sigmoid”}	“poly”
degree	{1, 2, 3, 4, 5}	2
gamma	{“scale”, “auto”}	“scale”
coef0	{0.0, 0.1, 0.2, 0.3, 0.4, 0.5}	0.2
C	{0.001, 0.01, 0.1, 1, 10, 100, 1000}	0.1
shrinking	{True, False}	True

**Table 4:** Hyperparameter optimization results for *k*-NN.

Hyperparameter	Search Range	Determined Value
n_neighbors	{2, 3, 4, 5, 6, 7, 8, 9, 10}	5
weights	{“uniform”, “distance”}	“uniform”
leaf_size	{10, 20, 30, 40, 50}	10
metric	{“minkowski”, “euclidean”, “cityblock”}	“minkowski”

**Table 5:** Hyperparameter optimization results for RF.

Hyperparameter	Search Range	Determined Value
n_estimators	{10, 20, 30, 40, 50, 60, 70, 80, 90, 100}	100
criterion	{“squared_error”, “absolute_error”, “friedman_mse”, “poisson”}	“absolute_error”
max_depth	{3, 4, 5, 6, 7, 8, 9, 10}	8

**Table 6:** Hyperparameter optimization results for DT.

Hyperparameter	Search Range	Determined Value
criterion	{“squared_error”, “friedman_mse”, “absolute_error”, “poisson”}	“friedman_mse”
splitter	{“best”, “random”}	“random”
max_depth	{3, 4, 5, 6, 7, 8, 9, 10}	8

**Table 7:** Hyperparameter optimization results for MLR.

Hyperparameter	Search Range	Determined Value
fit_intercept	{True, False}	True
copy_X	{True, False}	True
positive	{True, False}	False

**Table 8:** Hyperparameter optimization results for LASSO.

Hyperparameter	Search Range	Determined Value
alpha	{0.001, 0.01, 0.1, 1, 10, 100}	0.1
fit_intercept	{True, False}	True
copy_X	{True, False}	True
warm_start	{True, False}	True
positive	{True, False}	False

**Table 9:** Hyperparameter optimization results for Ridge.

Hyperparameter	Search Range	Determined Value
alpha	{0.001, 0.01, 0.1, 1, 10, 100}	10
fit_intercept	{True, False}	True
copy_X	{True, False}	True
positive	{True, False}	False

**Table 10:** Hyperparameter optimization results for Elastic Net.

Hyperparameter	Search Range	Determined Value
alpha	{0.001, 0.01, 0.1, 1, 10, 100}	10
l1_ratio	{0.1, 0.2, 0.3, 0.4, 0.5, 0.6, 0.7, 0.8, 0.9}	0.1
fit_intercept	{True, False}	True
copy_X	{True, False}	False
warm_start	{True, False}	False
positive	{True, False}	False

**Table 11:** Hyperparameter configuration for ANNs.

Reference	# Neurons in the 1st Hidden Layer	# Neurons in the 2nd Hidden Layer	Activation Function
[38]	7	3	tanh
[63]	10	10	tanh
[65]	80	None	tanh
[68]	9	None	tanh
[70]	4	None	tanh
[71]	10	None	sigmoid
[72]	3	None	sigmoid
[73,75]	20	None	sigmoid
[74]	57	None	sigmoid

**Table 12:** Performance comparison between ensemble models with different numbers of ANNs.

# ANNs (*T*)	MSE	RMSE	MAE	MAPE	MSLE	RMSLE	$R^{2}$
4	25.4125	5.0411	3.1862	0.0229	0.0013	0.0362	0.6918
8	22.6888	4.7633	2.9207	0.0209	0.0012	0.0342	0.7248
12	17.3473	4.1650	2.2916	0.0163	0.0009	0.0298	0.7896
16	13.1180	3.6219	1.7190	0.0123	0.0007	0.0260	0.8409
20	8.6529	2.9416	1.2753	0.0090	0.0004	0.0210	0.8951
24	9.3429	3.0566	1.3956	0.0099	0.0005	0.0219	0.8867
28	9.0502	3.0084	1.3400	0.0095	0.0005	0.0215	0.8902
32	10.0002	3.1623	1.4605	0.0104	0.0005	0.0226	0.8787
36	9.4920	3.0809	1.4201	0.0101	0.0005	0.0221	0.8849
40	9.7920	3.1292	1.4149	0.0101	0.0005	0.0224	0.8812

**Table 13:** Performance comparison between the proposed and benchmark methods.

Method	MSE	RMSE	MAE	MAPE	MSLE	RMSLE	$R^{2}$
SVM	59.2186	7.6954	5.3799	0.0397	0.0032	0.0569	0.2818
*k*-NN	11.7490	3.4277	2.4983	0.0178	0.0006	0.0248	0.8575
RF	20.0194	4.4743	3.1856	0.0228	0.0010	0.0320	0.7572
DT	22.4978	4.7432	3.5409	0.0253	0.0012	0.0340	0.7271
MLR	60.9421	7.8065	5.8778	0.0427	0.0033	0.0571	0.2609
LASSO	62.0635	7.8780	5.9153	0.0430	0.0033	0.0576	0.2473
Ridge	61.0068	7.8107	5.8782	0.0428	0.0033	0.0572	0.2601
ElasticNet	80.6553	8.9808	6.6842	0.0488	0.0043	0.0655	0.0218
[38]	82.7867	9.0987	6.7748	0.0495	0.0044	0.0663	−0.0040
[63]	51.1670	7.1531	5.3816	0.0387	0.0027	0.0516	0.3794
[65]	53.2452	7.2969	5.5224	0.0401	0.0028	0.0532	0.3542
[68]	45.5829	6.7515	5.0839	0.0367	0.0024	0.0487	0.4472
[70]	66.3351	8.1446	5.9073	0.0430	0.0035	0.0591	0.1955
[71]	48.0863	6.9344	5.2745	0.0380	0.0025	0.0501	0.4168
[72]	64.6372	8.0397	5.8128	0.0423	0.0034	0.0583	0.2161
[73,75]	51.1208	7.1499	5.4451	0.0394	0.0027	0.0518	0.3800
[74]	60.2464	7.7619	5.8966	0.0428	0.0032	0.0567	0.2693
Proposed	8.6529	2.9416	1.2753	0.0090	0.0004	0.0210	0.8951
